# Integrated analysis of anti-tumor roles of BAP1 in osteosarcoma

**DOI:** 10.3389/fonc.2022.973914

**Published:** 2022-08-08

**Authors:** Dong Hu, Yongbin Zheng, Xuehai Ou, Lijun Zhang, Xiaolong Du, Shaoyan Shi

**Affiliations:** ^1^ Depertment of Hand Surgery, Honghui Hospital, Xi’an Jiaotong University, Xi'an, China; ^2^ Peking University, Beijing, China; ^3^ Target Discovery Institute, Nuffield Department of Medicine, University of Oxford, Oxford, United Kingdom; ^4^ University of Auckland, Oakland, New Zealand

**Keywords:** GEO, target, osteosarcoma, BAP1, immune infiltration, EMT

## Abstract

**Background:**

This study aims to screen out differentially expressed genes (DEGs) regulated by BRCA1-associated protein 1 (BAP1) in osteosarcoma cells, and to analyze their biological functions.

**Methods:**

The microarray dataset GSE23035 of BAP1-knockdown osteosarcoma cells was obtained from Gene Expression Omnibus (GEO) database, consisting of shControl, shBAP1#1 and shBAP1#2 samples. The DEGs between the BAP1-knockdown osteosarcoma cells and the untreated osteosarcoma cells were screened with limma package, and then subjected to Gene Ontology (GO) and Kyoto Encyclopedia of Genes and Genomes (KEGG) enrichment analysis. Gene Set Enrichment Analysis (GSEA) was also performed for the three groups of samples. Hub genes in a protein-protein interaction (PPI) network of DEGs was filtered, and then subjected to prognostic analysis and correlation analysis with BAP1 in Therapeutically Applicable Research to Generate Effective Treatments (TARGET) database. Besides, the correlation between BAP1 and biological processes/pathways was analyzed by Gene Set Variation Analysis (GSVA) method and the correlation between BAP1 and immune infiltration by CIBERSORT and ESTIMATE methods. The roles of BAP1 in regulating proliferation and epithelial-mesenchymal transition (EMT) were validated by CCK-8 and western blot.

**Results:**

58 upregulated DEGs and 81 downregulated DEGs were obtained with |logFC| ≥ 1 and adj.p < 0.05. Cell cycle, DNA repair, and focal adhesion were associated with BAP1 in datasets. Further, BAP1 was negatively correlated with naïve CD4 T cells infiltration. *In vitro*, BAP1 inhibited proliferation and EMT.

**Conclusion:**

BAP1 might be a tumor suppressor in osteosarcoma and a promising therapeutic target.

## Introduction

Osteosarcoma is the most common malignant bone tumor and has become the second leading cancer-related mortality factor in children and adolescents (1). The psychological toll of traditional surgical treatment for osteosarcoma patients is huge, and the majority of amputation patients die within 1 year of diagnosis (2). With the continuous improvement in the treatment of osteosarcoma, neoadjuvant chemotherapy combined with surgery has improved the overall survival rate, but the 5-year survival rate is still less than 70% (3). In addition, the recurrence and metastasis rates of osteosarcoma are high (4), and the survival time is significantly shorter for patients with recurrence, metastasis and chemotherapy resistance (5), so the search for new treatments has become a research priority. Recent advances in research on tumor-related signaling pathways and novel gene targeted therapies (6) have provided new strategies and approaches for the prevention and treatment of osteosarcoma.

BRCA1-associated protein 1 (BAP1) is a 729 amino acid deubiquitinating enzyme encoded by the BAP1 gene that removes ubiquitination modifications from substrate proteins, allowing the substrate to escape the “ubiquitin-proteasome” degradation pathway, enhancing its stability, or affecting the functional activity of the substrate, thereby regulating the relevant signaling (7). The encoded enzyme binds to BRCA1 protein through its ring finger domain and acts as a tumor suppressor (8). In addition, the enzyme may be involved in transcriptional regulation, cell cycle and growth regulation, response to DNA damage, and chromatin dynamics (9). Germline mutations in this gene may be associated with tumor predisposition syndrome (TPDS), which increases the risk of cancers, including malignant mesothelioma, uveal melanoma, and cutaneous melanoma (10). Currently, the relationship between BAP1 and tumors is receiving increasing attention, and its structural stability and functional integrity are of great importance to the performance of cancer suppression (8). However, the roles of BAP1 in osteosarcoma are currently unknown.

In recent years, multiple public databases have been widely used for diagnostic and prognostic biomarker studies of tumors, where microarray technology plays an increasingly important role (11). Gene expression profiling databases are an important tool in medical oncology with important clinical applications (12, 13). With the study of a large amount of gene expression profile data and the application of gene microarray technology, it has been shown that differentially expressed genes (DEGs) are involved in a variety of biological processes, pathways and molecular functions (14, 15). In this study, we obtained the osteosarcoma dataset from the Therapeutically Applicable Research to Generate Effective Treatments (TARGET) database and the BAP1 knockdown dataset GSE23035 associated with osteosarcoma from the gene expression omnibus (GEO) database. Moreover, we used bioinformatics to screen for potential BAP1-regulated hub genes in osteosarcoma and to analyze their immune infiltration patterns, providing new directions for the study of the pathogenesis and therapeutic strategies of osteosarcoma.

## Methods

### Data collection and preprocessing

The microarray dataset GSE23035 of human osteosarcoma cell line U20S was downloaded from the GEO database *via* the GEOquery package (16). U2OS cells, transfected with a non-target control shRNA (shControl) or shRNAs targeting BAP1 (shBAP1#1 and shBAP1#2), were selected with puromycin containing medium and then synchronized at the G1/S border to allow comparative analysis of gene expression. The probe information in the dataset was annotated with the platform file GPL570. Probes corresponding to more than one gene are removed, and only the probe with the largest signal value is retained in cases where there are multiple probes corresponding to the same gene in the dataset. Missing values in the dataset are added by interpolation, and then the data are normalized by the normalize Between Arrays function of the limma package. The inter-group clustering of samples is viewed by principal component analysis (PCA) plots. Examination of the interference efficiency of BAP1 showed that BAP1 expression was significantly lower in the shBAP1#1 and shBAP1#2 groups compared to the shControl group, indicating that BAP1 interference was effective.

From TARGET database, we downloaded mRNA-seq data of Counts format and corresponding clinical data in the osteosarcoma project. Then, the mRNA-seq data were log-transformed as TPM format and median centered.

### DEGs screening

The limma package was used to perform differential analysis. DEGs were filtered with |logFC| ≥ 1 and adj.*p* < 0.05 and visualized by volcano plots. DEGs common to the shBAP1#1 and shBAP1#2 groups was identified by a venn diagram and visualized by heatmaps *via* ComplexHeatmap package (2.2.0) (17).

### Gene ontology and kyoto encyclopedia of genes and genomes analysis

The GO functional enrichment analysis and KEGG pathway enrichment analysis were performed using the cluster Profiler package (3.14.3) (18) of R software. GO annotations are divided into three categories, including biological process (BP), cellular components (CC), and molecular function (MF). In the enrichment analysis, Fisher’ exact test was used to test whether the DEGs were enriched in a term, and p.adj<0.05 and qvalue<0.2 were set as the screening conditions. Besides, pearson analysis was used to evaluate the correlation of BAP1 and genes in enriched terms.

### Gene set enrichment analysis

GO-BP gene sets and KEGG pathway gene sets were obtained from The Molecular Signatures Database (MSigDB) (19) as the reference gene set. The enrichment of two BAP1-knockdown groups in the GSE23035 dataset relative to the shControl group was analyzed by GSEA (20). A false discovery rate (FDR) < 0.25 and adj.p<0.05 were considered statistically significant. Top 5 enriched gene sets with largest |NES| were illustrated.

### Protein-protein interaction

The 139 DEGs were submitted to STRING 11.5 database (21) for protein-protein interaction (PPI) network construction with an interaction score = 0.15. The results were imported into Cytoscape software (3.7.1), and the most significant network module was screened by the Molecular complex detection (MCODE) plug-in. The parameters were set as follows: degree-cutoff=2, node score cutoff=0.2, k-core=2, max. depth=100. In addition, the top 10 hub genes in the PPI network were screened using the between, closeness, and degree algorithms in the CytoHubba plug-in. The distribution of the hub genes in chromosomes was analyzed *via* RCircos package (22). The expression of hub genes was verified in three groups of GSE23035 samples.

### The expression and prognostic value of hub genes in TARGET database

The hub genes expression was validated in osteosarcoma dataset of the TARGET database. Then, the correlation of BAP1 and hub genes was analyzed *via* pearson method. Additionally, samples with missing prognostic data were excluded and Kaplan-Meier survival curves were plotted with Cox regression methods. *p*<0.05 was considered a statistically significant difference.

### Gene set variation analysis

We first downloaded the c2.cp.kegg.v7.4.symbols.gmt and c5.go.bp.v7.4.symbols.gmt subsets from MSigDB. Setting the minimum gene set to 5 and the maximum gene set to 5000. The enrichment score of each sample in each gene set was calculated with GSVA package (1.40.1) (23) using osteosarcoma dataset of the TARGET database. Besides, the samples were divided into low and high BAP1 groups. The difference of GSVA enrichment scores between BAP1 groups was filtered with |logFC|≥1.2 and adj.*p*<0.05 and illustrated in a volcano plot.

### Immune infiltration in osteosarcoma tissue samples in the TARGET database

The immune infiltration scores in each sample from osteosarcoma dataset in the TARGET database were characterized with Estimation of Stromal and Immune cells in Malignant Tumors using Expression data (ESTIMATE) (24) and Cell-type Identification by Estimating Relative Subsets of RNA Transcripts (CIBERSORT) (25) algorithms. Besides, differential analysis and Pearson analysis were used to clarify the relationship of BAP1 and immune scores.

### Cell lines and transfection

Nontumor osteoblast cell line hFOB1.19 (CL-0353, Procell) was cultured using DMEM/F12 medium containing 10% FBS at 34°. Osteosarcoma cell lines SAOS2 (CL-0202, Procell) was cultured using McCoy’s 5A medium containing 10% FBS at 37°. Osteosarcoma cell lines SJSA1 (CL-0703, Procell) was cultured using RPMI-1640 medium containing 10% FBS at 37°. They were all cultured in an incubator containing 5% CO_2_.

To establish stable cell lines, the shRNAs targeting BAP1 (BAP1-sh1 and BAP1-sh2) and scrambed shRNA (NC-sh) were synthesized by Genechem (Shanghai, China) and cloned into the vector pLKO.1. The shRNAs sequences were shown in **Supplementary Table 1**. Besides, full-length BAP1 was inserted into pEF-HA vectors. According to the Lipofectamine 2000 (Invitrogen, Thermo Fisher Scientific, Inc.) instructions, the lentiviral vector carrying BAP1 or shRNA was transfected into SJSA1 cells or SAOS2 cells, respectively.

### qPCR

Total RNAs in cells were extracted by TRIzol reagent (Invitrogen). Reverse transcription was performed with ReverTra Ace kit (TOYOBO) at 37° for 15 min and 85° for 5 s. Quantitative PCR was performed with SYBR Premix Ex Taq (Takara) in conditions of 45 cycles of PCR, 95° for 30 s, 60° for 30 s, and 72° for 40s. GAPDH was served as an internal reference and the relative expression of BAP1 was calculated by 2-ΔΔCt method. The primers were listed in **Supplementary Table 2**.

### Western blot

The proteins from cells were extracted by RIPA buffer (Gibco), quantified *via* bicinchoninic acid (BCA) method, separated by 10% SDS-PAGE gel electrophoresis, and blotted onto PVDF membranes (Millipore). Then, the membranes were blocked with 5% skimmed milk for 1h and incubated overnight at 4° with primary antibody, including rabbit anti-BAP1 (1:1000, #13271, CST), rabbit anti-PCNA (1:1000, #13110, CST), rabbit anti-Vimentin (1:1000, #5741, CST), rabbit anti-E-Cadherin (1:1000, #3195, CST), rabbit anti-N-Cadherin (1:1000, #13116, CST), rabbit anti-GAPDH (1:1000, #2118, CST). Then, the membranes were incubated with goat anti-rabbit secondary antibody (1:1000, A0208, Beyotime) for 1h at room temperature. The blots were visualized with BeyoECL Star kit (P0018AM, Beyotime).

### Cell counting kit-8

Cell suspensions were prepared and inoculated into 96-well plates with approximately 2×10^3^ cells/100 μL/well. Three replicate wells were set up for each group. After 24, 48 and 72 h of culture, each well was added with 10 μL of CCK8 solution (C0038, Beyotime, China) and incubated for 2 h at 37°C. The absorbance values of each well were measured at 450 nm.

### Statistical analysis

All experimental results were expressed as mean ± standard deviation, and GraphPad Prism 9 (GraphPad Software, La Jolla, CA, USA) was used for statistical analysis. The mean between two groups was compared by independent sample t-test, and the mean between multiple groups was compared by one-way ANOVA analysis or two-way ANOVA analysis, and the difference was considered statistically significant at *p* < 0.05.

## Results

### DEGs obtaining

**Figure 1:**
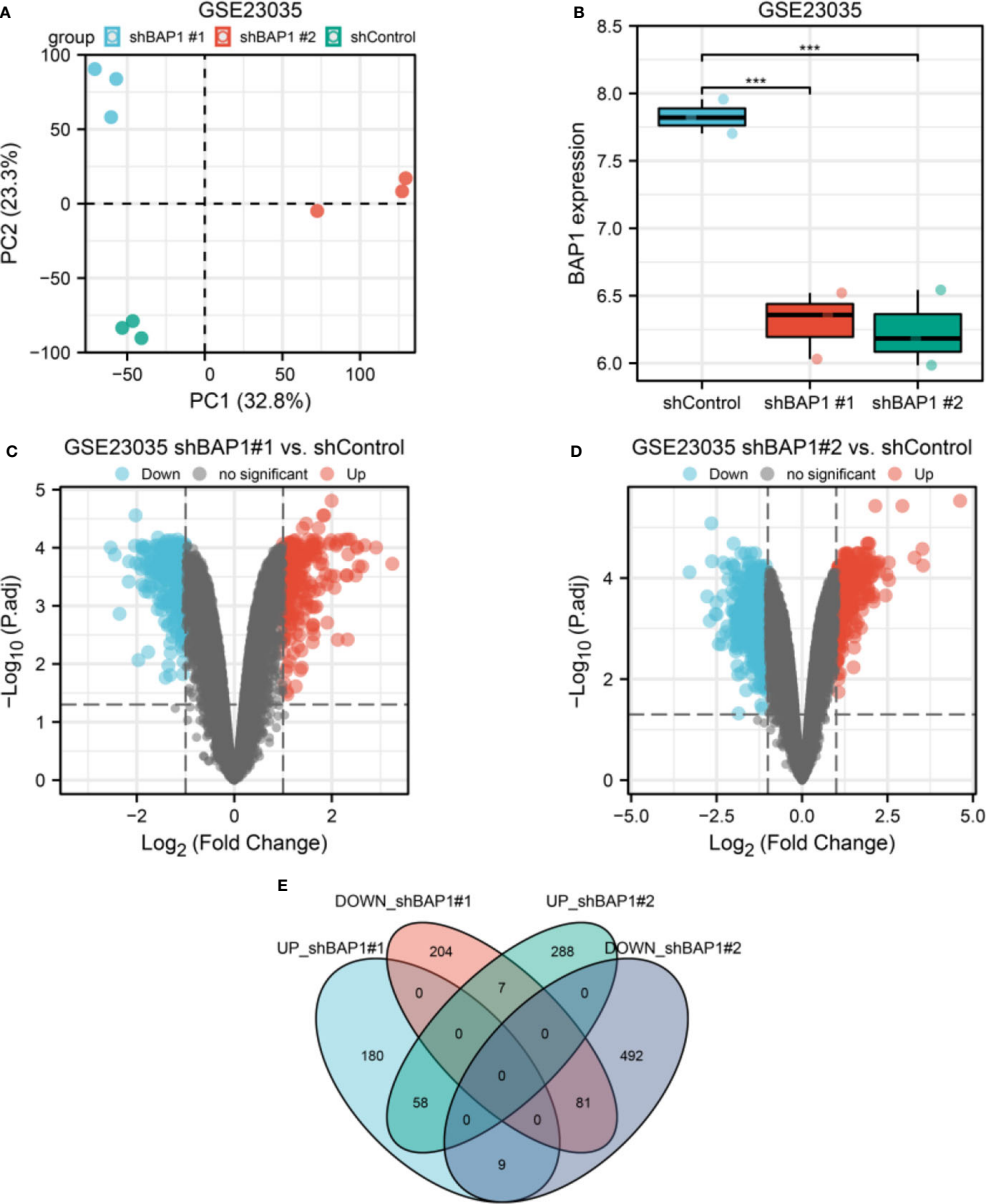
DEGs screening in GSE23035. **(A)** PCA plot of three groups of osteosarcoma cell samples in GSE23035. **(B)** BAP1 expression in shControl, shBAP1#1 and shBAP1#2 samples. **(C)** A volcano plot showing DEGs between shControl and shBAP1#1 groups. **(D)** A volcano plot showing DEGs between shControl and shBAP1#2 groups. **(E)** Overlapped DEGs between shBAP1#1 and shBAP1#2 were screened *via* a venn diagram ***p < 0.001.

**Figure 2:**
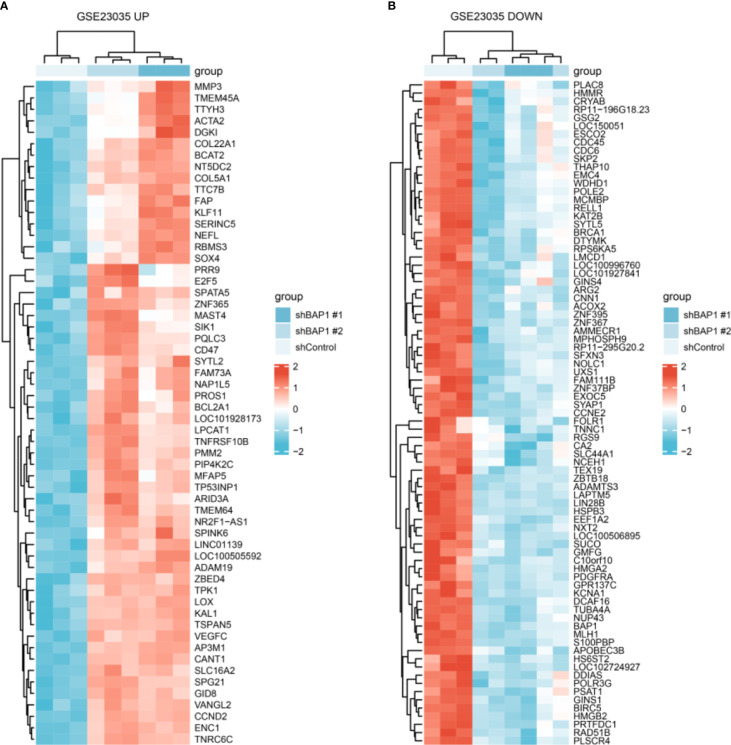
DEGs expression in shControl, shBAP1#1 and shBAP1#2 samples. **(A)** Upregulated DEGs. **(B)** Downregulated DEGs.

PCA plot showed well clustering of three groups of samples (**Figure 1A**). BAP1 expression was both downregulated in shBAP1#1 and shBAP1#2 groups (**Figure 1B**), suggesting the model samples were well constructed. Then, the DEGs were screened (**Figures 1C–E**) and demonstrated in **Figure 2**.

### Enrichment analysis

**Figure 3:**
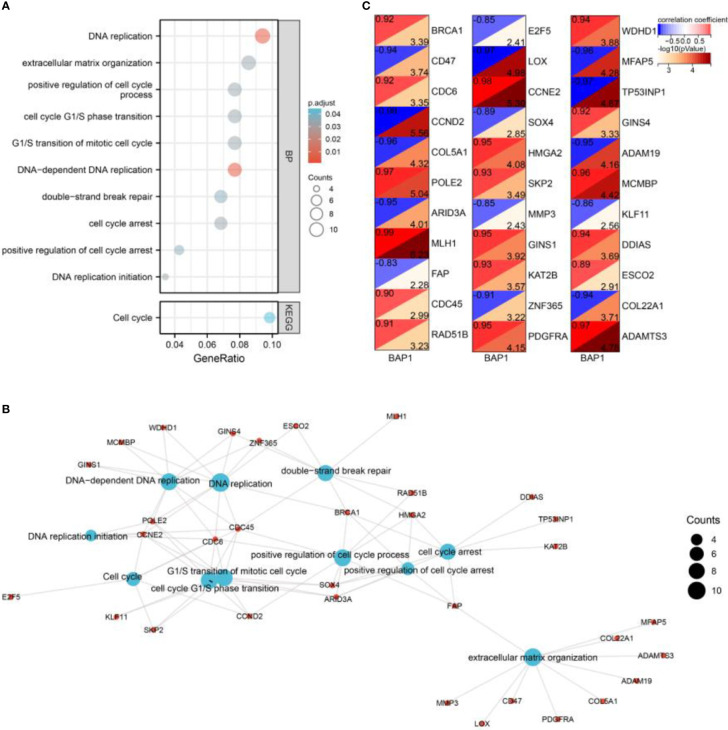
Enrichment analysis of DEGs. **(A)** GO and KEGG analysis of DEGs. **(B)** A network of enriched terms and DEGs. **(C)** Pearson analysis of BAP1 and DEGs enriched in GO and KEGG terms.

By GO and KEGG analysis, we found that DEGs were enriched in DNA replication, DNA-dependent DNA replication, DNA replication initiation, extracellular matrix organization, G1/S transition of mitotic cell cycle, cell cycle G1/S phase transition, positive regulation of cell cycle process, cell cycle arrest, positive regulation of cell cycle arrest, Cell cycle, and double-strand break repair (**Figures 3A, B**). Besides, the genes in enriched terms were all correlated with BAP1 (**Figure 3C**).

**Figure 4:**
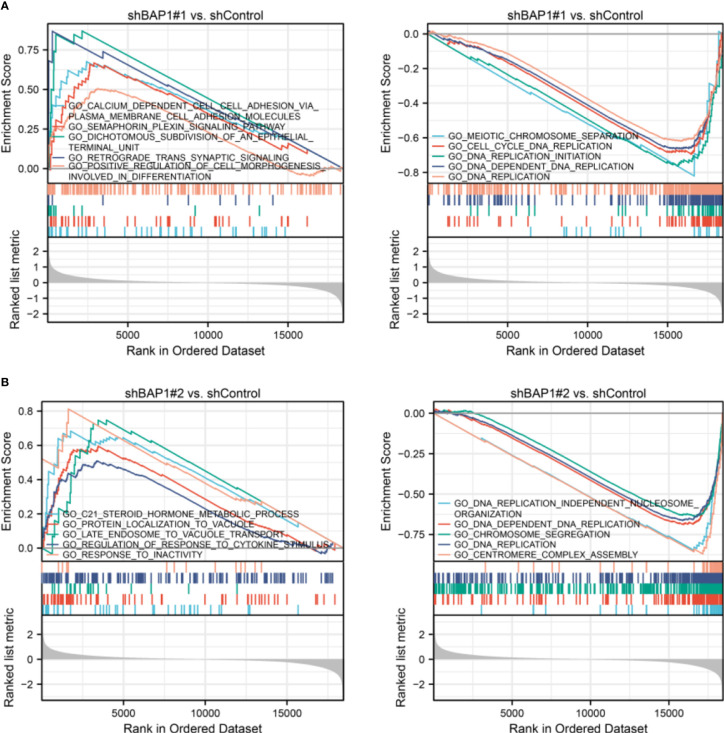
GSEA analysis in GO gene sets. **(A)** Top 5 GO terms with largest |NES| enriched in shBAP1#1 samples. **(B)** Top 5 GO terms with largest |NES| enriched in shBAP1#2 samples.

**Figure 5:**
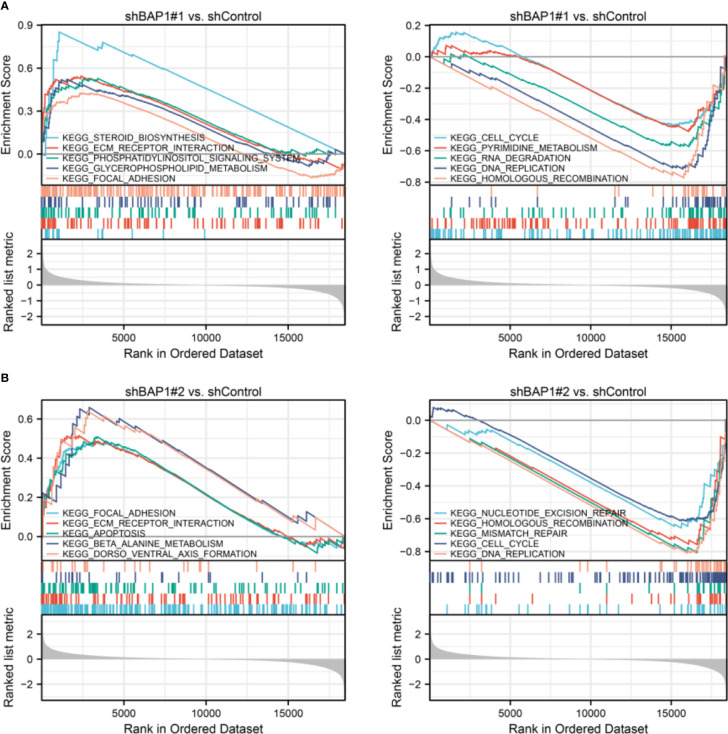
GSEA analysis in KEGG gene sets. **(A)** Top 5 KEGG terms with largest |NES| enriched in shBAP1#1 samples. **(B)** Top 5 KEGG terms with largest |NES| enriched in shBAP1#2 samples.

In terms of GSEA analysis, DNA replication-, extracellular matrix organization-, and cell cycle- related terms were also enriched. Top 5 GO terms with largest |NES| enriched in shBAP1#1 and shBAP1#2 samples were presented in **Figure 4**. Top 5 KEGG terms with largest |NES| enriched in shBAP1#1 and shBAP1#2 samples were presented in **Figure 5**. Interestingly, focal adhesion was enriched in shBAP1#1 and shBAP1#2 samples (**Figure 5**), suggesting BAP1 might inhibit EMT in osteosarcoma.

### Hub genes screening

**Figure 6:**
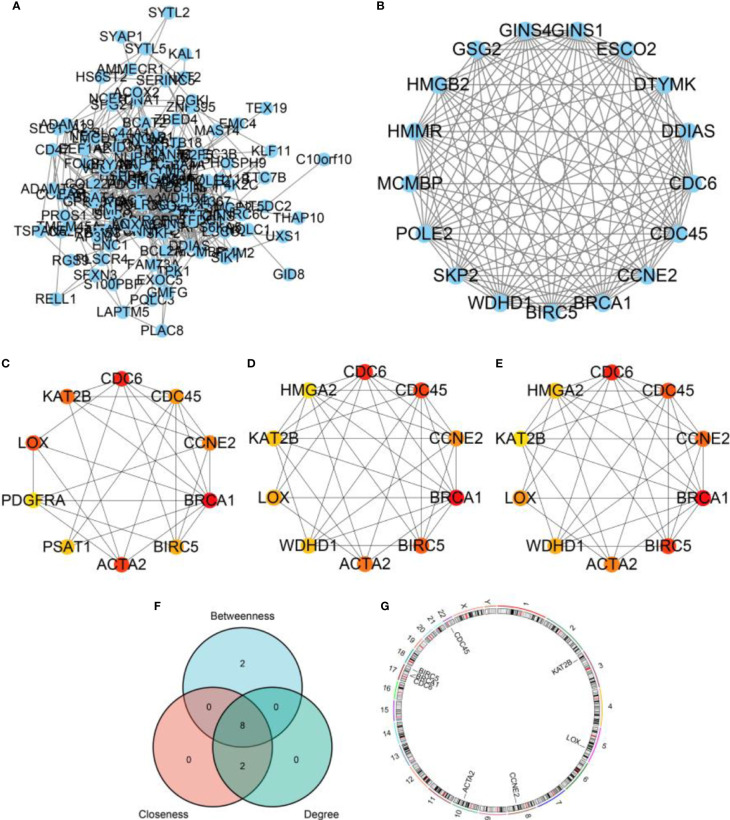
PPI network and hub genes. **(A)** A PPI network based on DEGs. **(B)** The most significant cluster found by MCODE plugin in Cytoscape software. **(C)** Hub genes calculated by Between algorithm. **(D)** Hub genes calculated by Closeness algorithm. **(E)** Hub genes calculated by Degree algorithm. **(F)** A venn diagram displaying overlapped hub genes. **(G)** The distribution of overlapped hub genes in chromosomes.

By constructing PPI network of DEGs (**Figure 6A**), we found a most significant module (**Figure 6B**). Besides, through cytoHubba plugin, we calculated top 10 hub genes *via* between (**Figure 6C**), closeness (**Figure 6D**), and degree (**Figure 6E**) algorithms, respectively. Further, we obtained 8 overlapped hub genes by the three algorithms, including ACTA2, BIRC5, BRCA1, CCNE2, CDC45, CDC6, KAT2B, and LOX (**Figure 6F**). The distribution of the hub genes was plotted in **Figure 6G**.

### Hub genes expression and prognostic value

**Figure 7:**
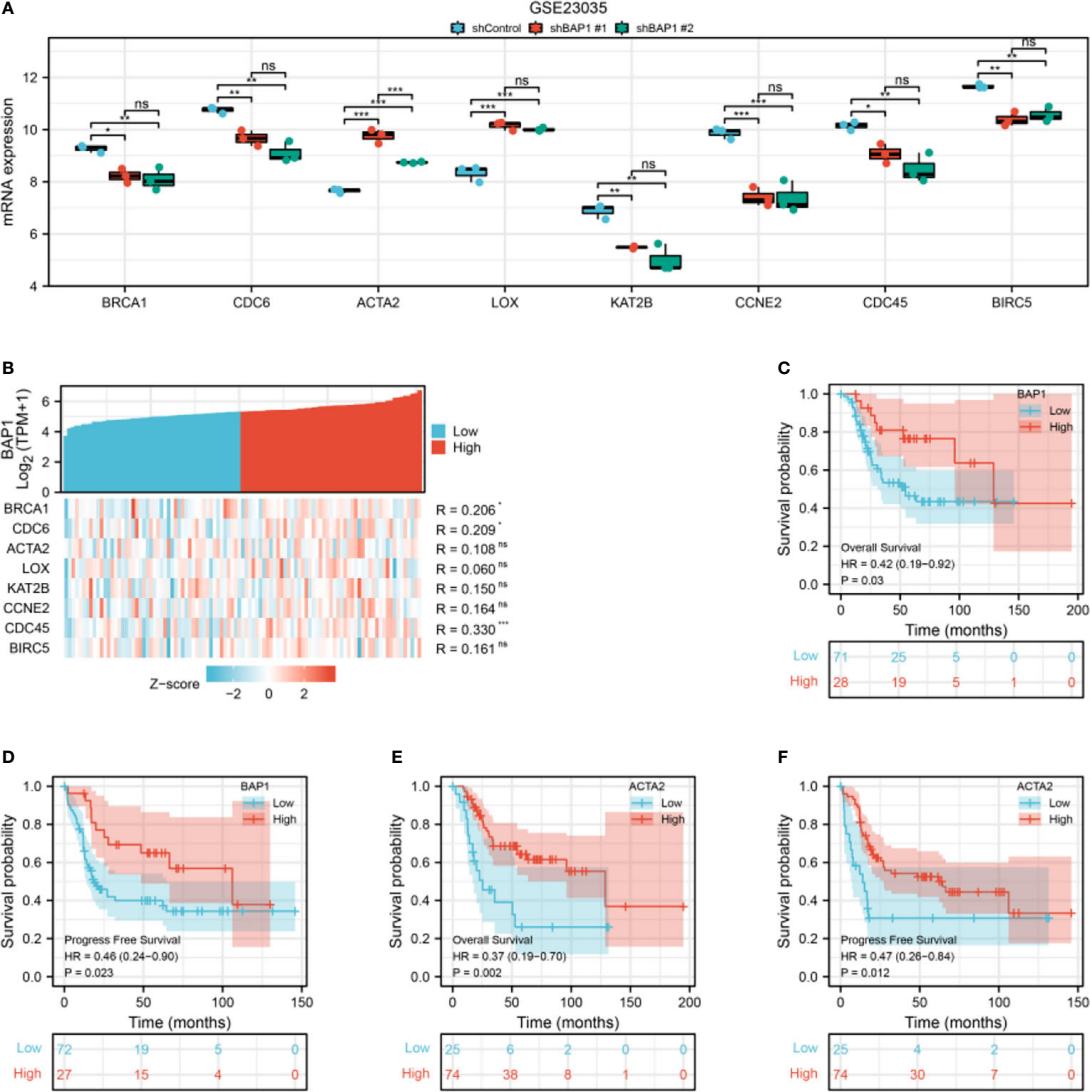
The expression and prognostic value of hub genes in GSE23035 and osteosarcoma dataset of TARGET database. **(A)** Hub genes expression in GSE23035. **(B)** The correlation of BAP1 and hub genes in the osteosarcoma dataset of TARGET database. **(C)** The overall survival of osteosarcoma patients in low and high BAP1 groups in TARGET database. **(D)** The progress free survival of osteosarcoma patients in low and high BAP1 groups in TARGET database. **(E)** The overall survival of osteosarcoma patients in low and high ACTA2 groups in TARGET database. F, The progress free survival of osteosarcoma patients in low and high ACTA2 groups in TARGET database. *p < 0.05; **p < 0.01; ***p < 0.01. ns, non significant.

Among hub genes, BRCA1, CDC6, KAT2B, CCNE2, CDC45, and BIRC5 were downregulated, while ACTA2 and LOX were upregulated in shBAP1#1 and shBAP1#2 samples in GSE23035 (**Figure 7A**). In the osteosarcoma dataset of TARGET database, although BAP1 expression was similar in osteosarcoma tissues from patients with different clinicopathologic characteristics (**Supplementary Table 3**), it was positively correlated with BRCA1, CDC6, and CDC45 (**Figure 7B**). Besides, low expression of BAP1 and ACTA2 was correlated with poor overall survival (**Figures 7C, E**) and progress free survival (**Figures 7D, F**). However, the expression of BIRC5, BRCA1, CCNE2, CDC45, CDC6, KAT2B, and LOX did not affect survival (**Figure S1**).

### GSVA analysis of the osteosarcoma dataset in TARGET database

**Figure 8:**
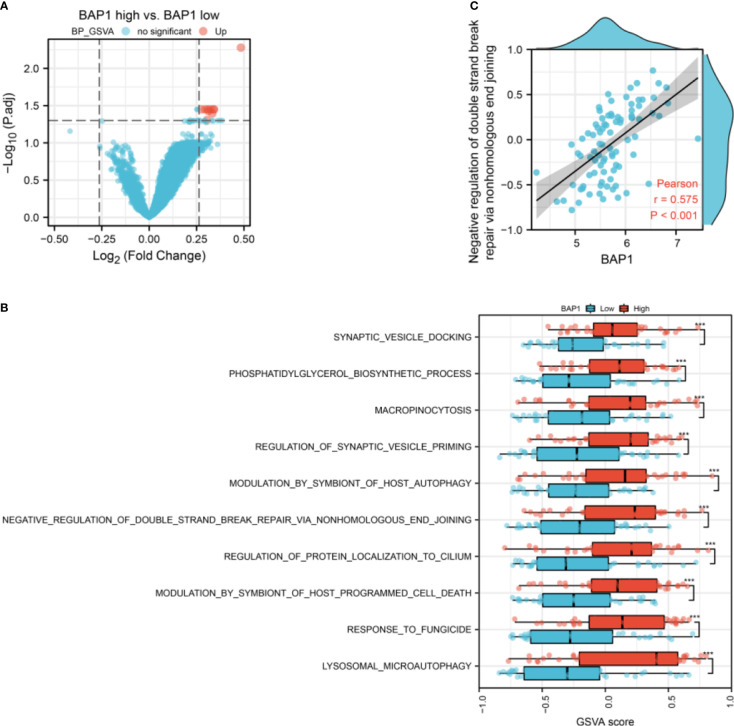
The correlation of BAP1 and GSVA score of GO-BP gene sets. **(A)** The difference of GSVA score of GO-BP gene sets in low and high BAP1 groups. **(B)** The GO-BP gene sets with differential GSVA score. **(C)** The correlation of BAP1 and negative regulation of double strand break repair *via* nonhomologous end joining.

To confirm the role of BAP1 in GSE23035, we performed GSVA analysis in the osteosarcoma dataset in TARGET database. There were 10 differential terms in GO-BP gene sets between high and low BAP1 groups (**Figure 8A**). In high BAP1 group, the GSVA score of lysosomal micro autophagy, response to fungicide, modulation by symbiont of host programmed cell death, regulation of protein localization to cilium, negative regulation of double strand break repair *via* nonhomologous end joining, modulation by symbiont of host autophagy, regulation of synaptic vesicle priming, micropinocytosis, phosphatidylglycerol biosynthetic process, and synaptic vesicle docking was higher than that in low BAP1 group (**Figure 8B**). Consistent with above results in GSE23035, BAP1 was positively correlated with negative regulation of double strand break repair *via* nonhomologous end joining (**Figure 8C**). In terms of KEGG gene sets, there were no differential terms (**Figure S2**).

### BAP1 was negatively correlated with naïve CD4 T cells infiltration

**Figure 9:**
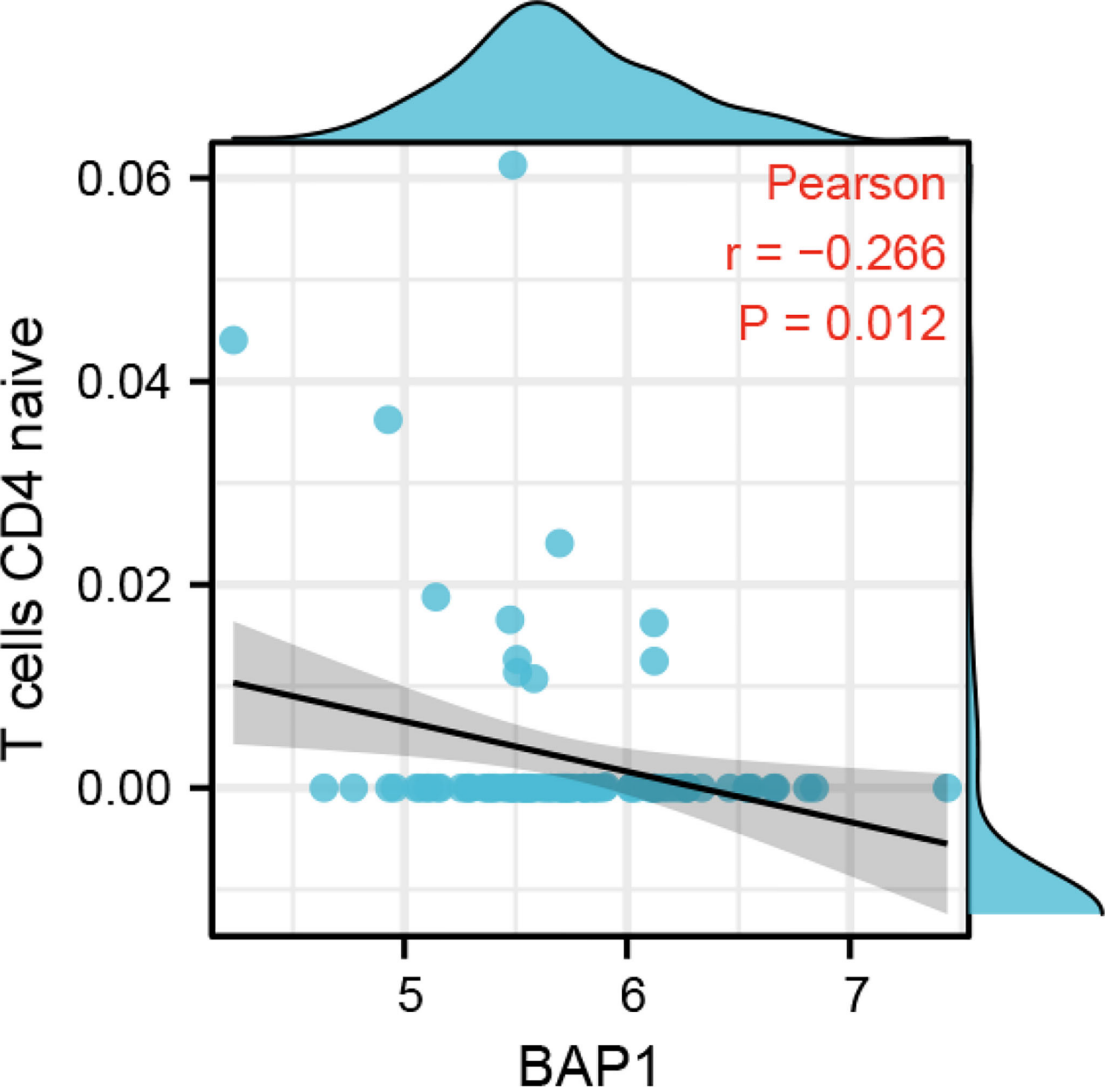
The correlation of BAP1 and naïve CD4 T cells infiltrated in osteosarcoma tissues.

To investigate the role of BAP1 in regulating immune infiltration, we used CIBERSORT and ESTIMATE methods. The results based on CIBERSORT algorithm showed that there was no difference in immune cell infiltration fraction between high and low BAP1 groups (**Figure S3A**). However, Pearson analysis demonstrated that BAP1 was negatively correlated with the fraction of naïve CD4 T cells in osteosarcoma tissues (**Figure 9**). In ESTIMATE algorithm, the results also displayed that BAP1 was not correlated with the fraction of stromal and other immune cell types (**Figures S3B–D**).

### BAP1 suppressed osteosarcoma cell growth and EMT

**Figure 10:**
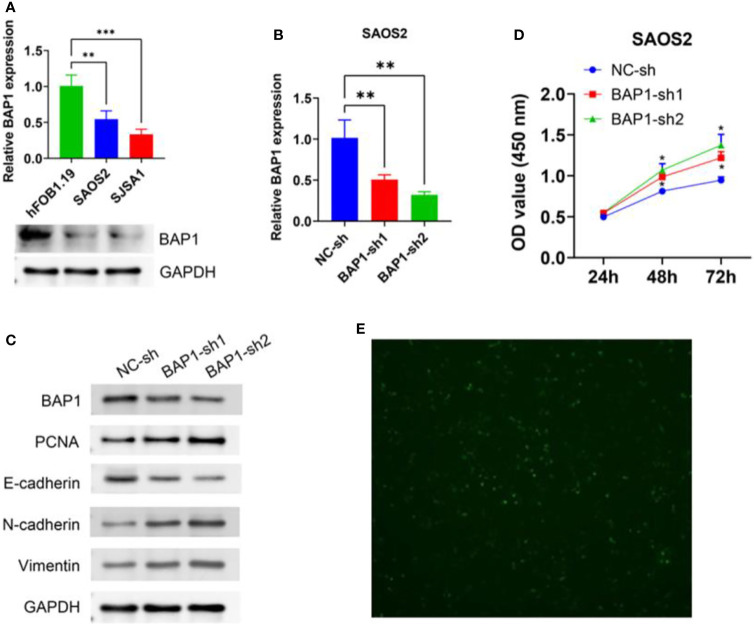
BAP1 knockdown promoted SAOS2 cells growth and EMT. **(A)** BAP1 expression in hFOB1.19, SAOS2, and SJSA1 cells detected by qPCR and western blot. **(B)** BAP1 expression after BAP1 knockdown detected by qPCR. **(C)** The protein expression of BAP1, PCNA, E-cadherin, N-cadherin, and Vimentin after BAP1 knockdown. **(D)** The viability of SAOS2 cells after BAP1 knockdown detected by CCK8. **(E)** Immunofluorescent image to detect BAP1 knockdown efficiency in SAOS2 cells. **p < 0.01.

**Figure 11:**
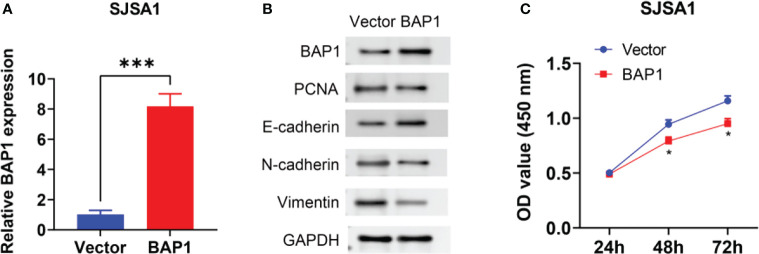
BAP1 overexpression inhibited SAOS2 cells growth and EMT. **(A)** BAP1 expression after BAP1 overexpression detected by qPCR. **(B)** The protein expression of BAP1, PCNA, E-cadherin, N-cadherin, and Vimentin after BAP1 overexpression. **(C)** The viability of SAOS2 cells after BAP1 overexpression detected by CCK-8. ***p < 0.001.

To further validate anti-cancer effects of BAP1, we first detected BAP1 expression in nontumor osteoblast cell line hFOB1.19 and osteosarcoma cell lines SAOS2 and SJSA1. The results showed that mRNA and protein expression of BAP1 was higher in hFOB1.19 cells than that in SAOS2 and SJSA1 cells (**Figure 10A**). Then we silenced or overexpressed BAP1 in SAOS2 and SJSA1 cells, respectively (**Figures 10B**, **11A**). In BAP1-knockdown SAOS2 cells, we found higher protein expression of PCNA, N-cadherin, and Vimentin than in control SAOS2 cells (**Figure 10C**). Further, higher growth rate was observed in BAP1-knockdown SAOS2 cells (**Figure 10D**). (**Figure 10E**) Immunofluorescent image to detect BAP1 knockdown efficiency in SAOS2 cells. In BAP1-overexpression SJSA1 cells, the opposite results displayed, implying BAP1 could inhibit osteosarcoma cell growth and EMT (**Figures 11B, C**).

## Discussion

Osteosarcoma has a high degree of malignancy, progresses rapidly, with the majority advanced at the time of diagnosis, and metastasizes easily to the lung (26). In this study, by analyzing osteosarcoma dataset GSE23035 in GEO database, we found that BAP1 could significantly regulate 139 genes expression. These DEGs were involved in DNA replication, cell cycle, and DNA repair. Among these DEGs, hub genes were ACTA2, BIRC5, BRCA1, CCNE2, CDC45, CDC6, KAT2B, and LOX. In TARGET database, low BAP1 or ACTA2 expression both predicted poor overall survival and progress free survival. Besides, BAP1 was negatively correlated with naïve CD4 T cells infiltration. *In vitro*, BAP1 could inhibit osteosarcoma cells proliferation and EMT.

BAP1 belongs to the ubiquitin C-terminal hydrolase subfamily of deubiquitinases (27). Most of the earlier studies demonstrated that BAP1 exerts a tumor suppressive function. Overexpression of BAP1 in breast cancer MCF-7 cells inhibits the formation of soft agar clones (28). Overexpression of wild-type BAP1 in lung cancer NCI-H226 cells significantly inhibits the tumorigenic ability of the cells in nude mice, while overexpression of BAP1 with either enzyme-activity mutation (C91A) or nuclear localization sequence deletion (NLS2-Ala) did not affect the tumorigenic ability (29), suggesting that the anti-tumor function of BAP1 depends on its catalytic activity and nuclear localization. However, in recent years, some contrary reports have also emerged. For example, one study showed that BAP1 promotes the development of breast cancer by enhancing the stability of the transcription factor KLF5 (30). In this study, we found that focal adhesion was enhanced in shBAP1 cell samples and negative regulation of double strand break repair *via* nonhomologous end joining was enhanced in BAP1 high tissue samples, suggesting its cancer-inhibiting effect. In addition, Lysyl oxidase (LOX) and its family members LOXL1-4, the copper-dependent amine oxidases playing critical roles in ECM crosslinking and remodeling, are implicated in cancer progression and metastasis. The transduction of resultant matrix mechanical property changes into cellular signaling promotes disruption of cell polarity, dynamic cytoskeleton rearrangement, cell migration and invasion (31). Furthermore, the acquisition of invasive behavior of cells expressing Alpha-Actin (ACTA2) are also partially attributed to the EMT in transcription factor snail dependent- and independent- manners (32). Therefore, the roles in anti-proliferation and anti-EMT should be further validated *in vitro*.

Mutations in BAP1 may affect the deubiquitinase activity of BAP1 protein or lead to deletion of its nuclear localization sequence (31), disrupting its anti-cancer function and ultimately causing tumorigenesis. Mutations in BAP1 were first identified in studies of familial malignancies, which manifested as increased prevalence of rare malignancies in some families, such as malignant mesothelioma, cutaneous melanoma, and uveal melanoma (32). However, we could not find any osteosarcoma dataset about BAP1 mutation, so the potential mechanisms involved in the progression of osteosarcoma are to be explored later. Additionally, Roy Baas et al. found by mass spectrometry analysis that BAP1 interacts with various proteins such as ASXL1, HAT1, COPI, etc (33). The underlying molecular mechanisms directly mediated by BAP1 in osteosarcoma need to be further explored.

Reportedly, BAP1 could regulate many tumors microenvironment. Using integrated analysis, the relationship between BAP1 and multiple immune checkpoints in pan-cancer was revealed (34). Carlos R Figueiredo et al. reported that loss of BAP1 expression in uveal melanoma contributed to an immunosuppressive microenvironment (35). Loss of BAP1 in mesothelioma correlates with an inflammatory tumor microenvironment characterized by immune checkpoint receptor activation and BAP1 status might predict ICI therapy benefit (36, 37). Unlike in most cancers, BAP1 had no effects on immune infiltration in osteosarcoma.

Although Shuming Gao et al. reported the suppressive effects on cancer of BAP1 in osteosarcoma (38), only *in vitro* cellular studies were performed. This shortcoming makes the study low clinical translational value. In this study, the roles of BAP1 in potential targets, biological functions, signaling pathways, and immune infiltration were comprehensively explored by mining the osteosarcoma datasets from GEO and TARGET databases using the rapidly developing bioinformatics in recent years.

In summary, through bioinformatics and *in vitro* assays, this study demonstrated that BAP1 was a tumor suppressor in osteosarcoma and provided new clues for osteosarcoma treatment such as BAP1-targeted therapy.

## Data availability statement

The original contributions presented in the study are included in the article/**Supplementary Material**. Further inquiries can be directed to the corresponding author.

## Author contributions

DH and YZ jointly designed the study and analyzed the data. XO, LZ, XD, SS collected the data. YZ designed and participated in all experiments. All authors contributed to the article and approved the submitted version.

## Conflict of interest

The authors declare that the research was conducted in the absence of any commercial or financial relationships that could be construed as a potential conflict of interest.

## Publisher’s note

All claims expressed in this article are solely those of the authors and do not necessarily represent those of their affiliated organizations, or those of the publisher, the editors and the reviewers. Any product that may be evaluated in this article, or claim that may be made by its manufacturer, is not guaranteed or endorsed by the publisher.

## Supplementary material

Supplementary Figure 1 The prognostic value of hub genes analyzed in osteosarcoma dataset of TARGET database. **(A)** The relationship of BIRC5 and overall survival. **(B)** The relationship of BIRC5 and progress free survival. **(C)** The relationship of BRCA1 and overall survival. **(D)** The relationship of BRCA1 and progress free survival. **(E)** The relationship of CCNE2 and overall survival. **(F)** The relationship of CCNE2 and progress free survival. **(G)** The relationship of CDC45 and overall survival. **(H)** The relationship of CDC45 and progress free survival. **(I)** The relationship of CDC6 and overall survival. **(J)** The relationship of CDC6 and progress free survival. **(K)** The relationship of KAT2B and overall survival. **(L)** The relationship of KAT2B and progress free survival. **(M)** The relationship of LOX and overall survival. **(N)** The relationship of LOX and progress free survival.

Supplementary Figure 2 The difference of GSVA score of KEGG gene sets in low and high BAP1 groups.

Supplementary Figure 3 The relationship of BAP1 and immune infiltration in osteosarcoma dataset of TARGET database. **(A)** 22 types of immune cells estimated by CIBERSORT algorithm. **(B)** The correlation of BAP1 and StromalScore. **(C)** The correlation of BAP1 and ImmuneScore. **(D)**, The correlation of BAP1 and ESTIMATEScore.

Supplementary Table 1 Sequences of shRNA Against BAP1

Supplementary Table 2 Sequences of PCR primers

Supplementary Table 3 Correlation between BAP1 expression and clinicopathologic characteristics of osteosarcoma patients
