# Supplementary figures and images for: Integrated analysis of anti-tumor roles of BAP1 in osteosarcoma
Source: Front Oncol. 2022 Aug 8;12:973914. doi: 10.3389/fonc.2022.973914 (PMC9393745; doi:10.3389/fonc.2022.973914)

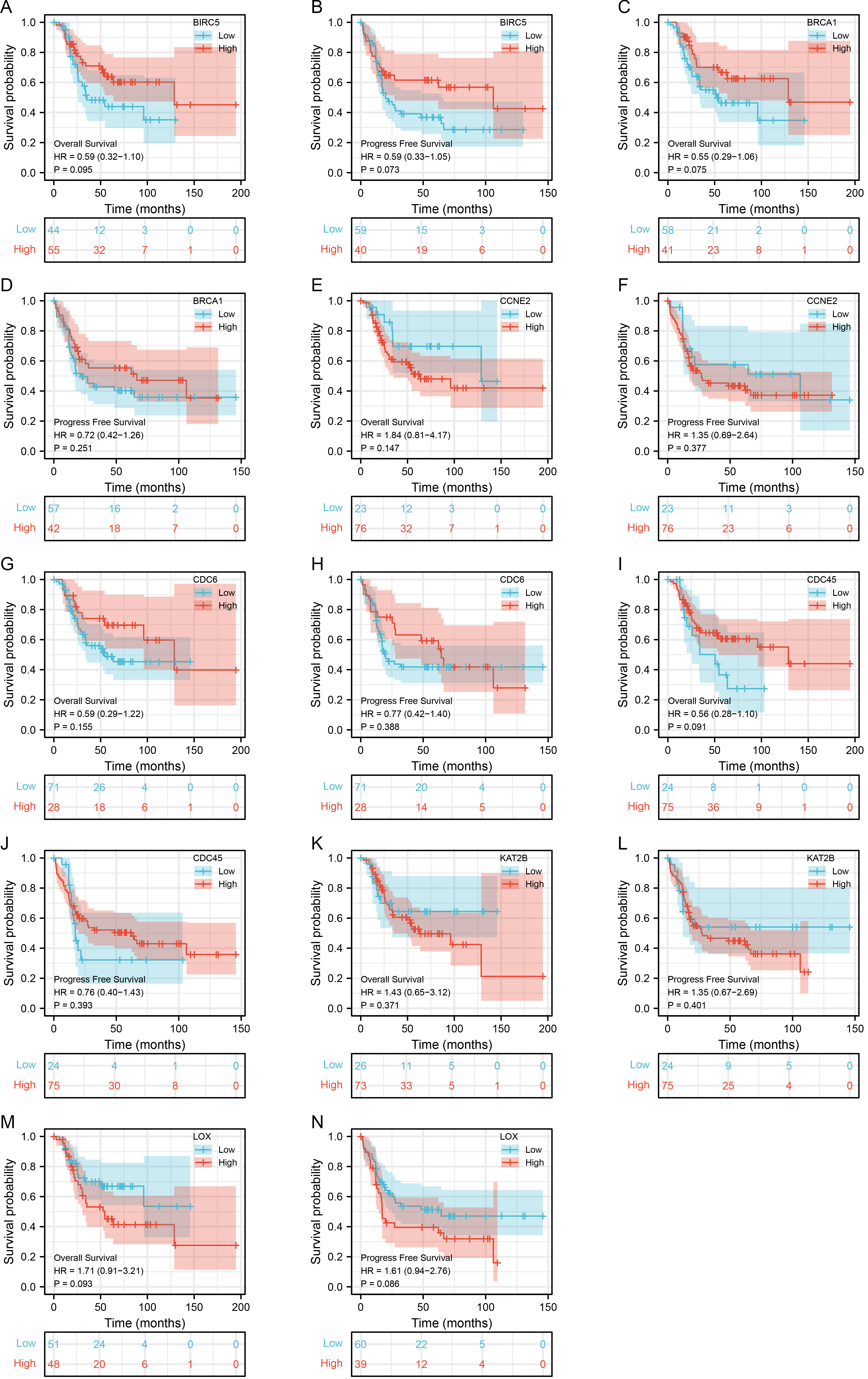

Supplement: Supplementary Figure 1 — The prognostic value of hub genes analyzed in osteosarcoma dataset of TARGET database. (A) The relationship of BIRC5 and overall survival. (B) The relationship of BIRC5 and progress free survival. (C) The relationship of BRCA1 and overall survival. (D) The relationship of BRCA1 and progress free survival. (E) The relationship of CCNE2 and overall survival. (F) The relationship of CCNE2 and progress free survival. (G) The relationship of CDC45 and overall survival. (H) The relationship of CDC45 and progress free survival. (I) The relationship of CDC6 and overall survival. (J) The relationship of CDC6 and progress free survival. (K) The relationship of KAT2B and overall survival. (L) The relationship of KAT2B and progress free survival. (M) The relationship of LOX and overall survival. (N) The relationship of LOX and progress free survival. [file Image_1.tif]

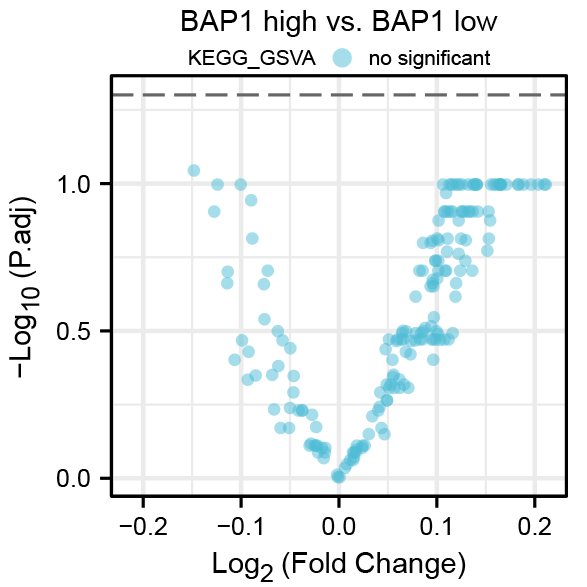

Supplement: Supplementary Figure 2 — The difference of GSVA score of KEGG gene sets in low and high BAP1 groups. [file Image_2.tif]

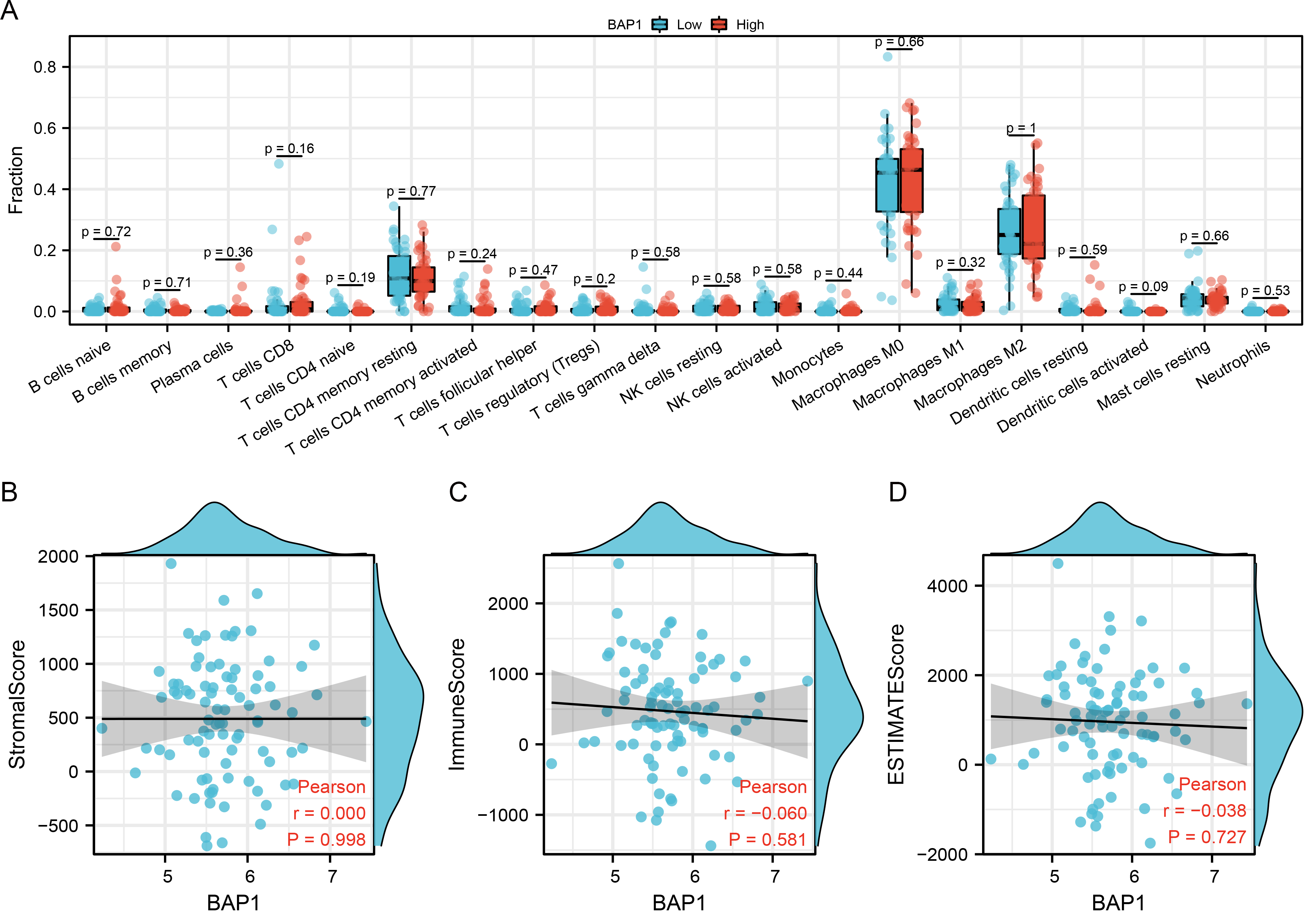

Supplement: Supplementary Figure 3 — The relationship of BAP1 and immune infiltration in osteosarcoma dataset of TARGET database. (A) 22 types of immune cells estimated by CIBERSORT algorithm. (B) The correlation of BAP1 and StromalScore. (C) The correlation of BAP1 and ImmuneScore. (D), The correlation of BAP1 and ESTIMATEScore. [file Image_3.tif]
