# Supplementary material for: Integrated analysis of anti-tumor roles of BAP1 in osteosarcoma
Source: Front Oncol. 2022 Aug 8;12:973914. doi: 10.3389/fonc.2022.973914 (PMC9393745; doi:10.3389/fonc.2022.973914)
Supplement: Supplementary Table 1 — Sequences of shRNA Against BAP1 [file Table_1.docx]

**Supplementary Table 1. Sequences of shRNA Against BAP1**

| NC-sh | 5’-3’ | CAACAAGATGAAGAGCACCAA |
| --- | --- | --- |
| BAP1-sh1 | 5’-3’ | ACAACTACGATGAGTTCAT |
| BAP1-sh2 | 5’-3’ | TGGAAGATTTCGGTGTCAA |
