# Supplementary material for: Integrated analysis of anti-tumor roles of BAP1 in osteosarcoma
Source: Front Oncol. 2022 Aug 8;12:973914. doi: 10.3389/fonc.2022.973914 (PMC9393745; doi:10.3389/fonc.2022.973914)
Supplement: Supplementary Table 2 — Sequences of PCR primers [file Table_2.docx]

**Supplementary Table 2. Sequences of PCR primers**

| BAP1 | Forward(5’-3’) | GACCCAGGCCTCTTCACC |
| --- | --- | --- |
|  | Reverse(5’-3’) | AGTCCTTCATGCGACTCAGG |
| GAPDH | Forward(5’-3’) | AGCCACATCGCTCAGACAC |
|  | Reverse(5’-3’) | GAATTTGCCATGGGTGGA |
