# Supplementary material for: Integrated analysis of anti-tumor roles of BAP1 in osteosarcoma
Source: Front Oncol. 2022 Aug 8;12:973914. doi: 10.3389/fonc.2022.973914 (PMC9393745; doi:10.3389/fonc.2022.973914)
Supplement: Supplementary Table 3 — Correlation between BAP1 expression and clinicopathologic characteristics of osteosarcoma patients [file Table_3.docx]

| Characteristic | Low expression of BAP1 | High expression of BAP1 | p |
| --- | --- | --- | --- |
| n | 50 | 51 |  |
| Metastasis, n (%) |  |  | 0.954 |
| No | 37 (36.6%) | 39 (38.6%) |  |
| Yes | 13 (12.9%) | 12 (11.9%) |  |
| Tumor region, n (%) |  |  | 0.835 |
| Distal | 20 (31.2%) | 16 (25%) |  |
| Other | 1 (1.6%) | 2 (3.1%) |  |
| Proximal | 14 (21.9%) | 11 (17.2%) |  |
| Proximal & Distal | 0 (0%) | 0 (0%) |  |
| Age, n (%) |  |  | 0.957 |
| <18 | 38 (37.6%) | 40 (39.6%) |  |
| >=18 | 12 (11.9%) | 11 (10.9%) |  |
| Gender, n (%) |  |  | 0.626 |
| Female | 22 (21.8%) | 19 (18.8%) |  |
| Male | 28 (27.7%) | 32 (31.7%) |  |
| Race, n (%) |  |  |  |
| American Indian or Alaska Native | 1 (1.3%) | 0 (0%) |  |
| Asian | 3 (3.9%) | 4 (5.3%) |  |
| Black or African American | 7 (9.2%) | 3 (3.9%) |  |
| Native Hawaiian or other Pacific Islander | 0 (0%) | 0 (0%) |  |
| White | 29 (38.2%) | 29 (38.2%) |  |
| Ethnicity, n (%) |  |  | 1.000 |
| Hispanic or Latino | 6 (8.3%) | 5 (6.9%) |  |
| Not Hispanic or Latino | 31 (43.1%) | 30 (41.7%) |  |
| Tumor side, n (%) |  |  | 0.707 |
| Left | 9 (33.3%) | 6 (22.2%) |  |
| Right | 6 (22.2%) | 6 (22.2%) |  |
| Primary site progression, n (%) |  |  | 0.321 |
| No | 16 (31.4%) | 16 (31.4%) |  |
| Yes | 13 (25.5%) | 6 (11.8%) |  |
| Surgery, n (%) |  |  |  |
| Amputation | 3 (5%) | 3 (5%) |  |
| Amputation and hemipvelvectomy | 0 (0%) | 0 (0%) |  |
| Limb sparing | 29 (48.3%) | 22 (36.7%) |  |
| Limb sparing; Amputation | 1 (1.7%) | 0 (0%) |  |
| No surgery | 2 (3.3%) | 0 (0%) |  |
| OS event, n (%) |  |  | 0.622 |
| Alive | 27 (27.3%) | 31 (31.3%) |  |
| Dead | 22 (22.2%) | 19 (19.2%) |  |
| PFS event, n (%) |  |  | 0.609 |
| No | 21 (21.2%) | 25 (25.3%) |  |
| Yes | 28 (28.3%) | 25 (25.3%) |  |
